# Expedited Care for a Patient With Uncertain Myocardial Infarction Using Point-of-Care Ultrasound in Critical Access Emergency Medicine: A Case Report

**DOI:** 10.7759/cureus.96828

**Published:** 2025-11-14

**Authors:** Jamal Jones, David M Crockett, Vismaya Kharkar, Sarah Petelinsek, Astrid Haaland, Jennifer Cotton

**Affiliations:** 1 Department of Emergency Medicine, University of Utah Health, Salt Lake City, USA; 2 Department of Internal Medicine, University of Utah Health, Salt Lake City, USA; 3 Department of Medicine, University of Utah Health, Salt Lake City, USA; 4 Department of Emergency Medicine, Univeristy of Utah Health, Salt Lake City, USA

**Keywords:** adult echocardiography, cardiac pocus, pocus, pocus in emergency medicine, point-of-care cardiac ultrasound, point-of-care-ultrasound, st-elevation myocardial infarction (stemi)

## Abstract

Point-of-care ultrasound (POCUS) is a valuable tool for emergency department (ED) physicians. In this case report, we demonstrate how the use of cardiac POCUS can enhance access to definitive care for emergent cardiac complaints in rural settings lacking the resources of tertiary care facilities. We present the case of a 73-year-old woman who presented to the ED with one hour of chest pain concerning for myocardial infarction versus acute pericarditis. Bedside echocardiography was performed, ruling out pericarditis and establishing the diagnosis. The rapid availability of POCUS reduced diagnostic uncertainty, enabling timely management of an ST-elevation myocardial infarction (STEMI), despite associated health risks and geographical barriers to immediate percutaneous coronary intervention. In order to reduce diagnostic uncertainty, bedside cardiac ultrasound was performed, with results most significant for STEMI. Ultimately, despite geographic barriers, as a result of POCUS, this patient was able to quickly access definitive care for a condition in which “time is tissue,” reducing both the potential morbidity and mortality associated with STEMIs.

## Introduction

Point-of-care ultrasound (POCUS) is a valuable tool for emergency department (ED) physicians. It is commonly used both in acute trauma settings and beyond to identify life-threatening conditions such as pericardial tamponade, ruptured abdominal aneurysm, or ruptured ectopic pregnancy. Cardiac POCUS, in particular, has many applications for the emergency department. In rural and critical-access settings, where radiology-performed echocardiography or cardiology consultation may be unavailable, cardiac POCUS serves as an essential extension of bedside assessment and a valuable clinical decision-making tool.

Several retrospective reviews have demonstrated that the use of cardiac POCUS leads to significantly shorter time-to-diagnosis and time-to-pericardiocentesis in patients with clinically significant pericardial effusions [1]. Moreover, cardiac POCUS can detect wall motion abnormalities, allowing for earlier identification of electrocardiographically occult occlusive myocardial infarction (OOMI) compared to standard evaluation. This early detection has the potential to expedite definitive management [2]. Additionally, cardiac POCUS is a valuable diagnostic tool in cardiac arrest protocols, providing key information about heart rhythm without disrupting resuscitation efforts [3]. Though echocardiographic findings such as regional wall-motion abnormalities are not specific to ischemia, they can be suggestive of the diagnosis in the appropriate clinical context. Because of this, POCUS should not be used to independently confirm or exclude myocardial infarction. Instead, it should be interpreted alongside EKG and clinical features to support stronger medical decision-making. 

Cardiac POCUS does not require extensive training for providers to identify key pathologies and can be considered a physical examination aid in some circumstances [4]. Although prior work has shown that POCUS can improve patient engagement and reduce imaging-related costs [5-7], the present report centers on its clinical and logistical role in time-sensitive cardiac emergencies within resource-limited environments.

This case report demonstrates the utility of cardiac POCUS in expediting diagnosis and management of acute cardiac emergencies within rural or critical-access hospitals. It also highlights a notable gap in existing literature: the limited documentation of POCUS-guided reperfusion decision-making for myocardial infarction when cardiology consultation or comprehensive echocardiography is unavailable.

## Case presentation

We report the case of a 73-year-old female patient who presented to a regional ED with a chief complaint of chest pain. The pain began acutely, at rest, approximately one hour prior to her arrival. She described the symptoms as chest pressure and associated it with dyspnea and mild diaphoresis. The pain was not positional, and she denied recent respiratory infections. 

She also endorsed one month of chest discomfort and shortness of breath when walking up a flight of stairs. However, she had a history of chronic hypoxic respiratory failure and was on intermittent home oxygen at two liters per minute. Two weeks prior to the presentation, she had been diagnosed with a deep vein thrombosis of the right lower extremity and was therapeutically anticoagulated with apixaban. She denied a cardiac history, hypertension, diabetes mellitus, hyperlipidemia, and tobacco use. 

Hospital information 

The presenting hospital serves as the regional medical center for a county of approximately 41,000 inhabitants. It also provides medical care to patients from neighboring counties. The hospital is located approximately 180 miles from the nearest tertiary care facility, which is also the nearest interventional cardiology facility. Transport to this facility takes 65 minutes by helicopter (under favorable weather conditions), 70 minutes via fixed-wing air transport (plus ambulance transport time), and three hours by ground transportation (without traffic). Patients often use privately owned vehicles for ground transport due to the costs associated with transportation. While helicopter transportation is the fastest, it is also the most expensive, with an estimated bill of approximately $50,000 from the presenting hospital to the nearest cath lab. 

There are no cardiologists available for on-site consultation or patient care at this hospital. Patients requiring cardiology consultation must either be transferred to another facility or receive care via telehealth. Additionally, the hospital lacks other formal echocardiography resources. 

ED course 

**Figure 1:**
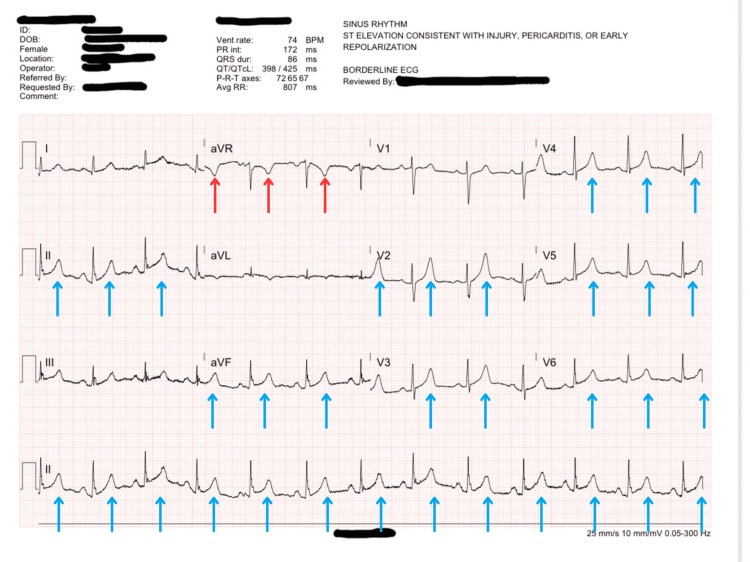
A 12-lead ECG obtained from the patient at the time of arrival showing ST-segment elevations in all precordial leads, lead II, and aVF. This raised concern for acute coronary syndrome. Additionally, this ECG demonstrates ST-depression in aVR. The ST-segment elevations are highlighted in blue, and the ST-segment depressions are in red.

**Figure 2:**
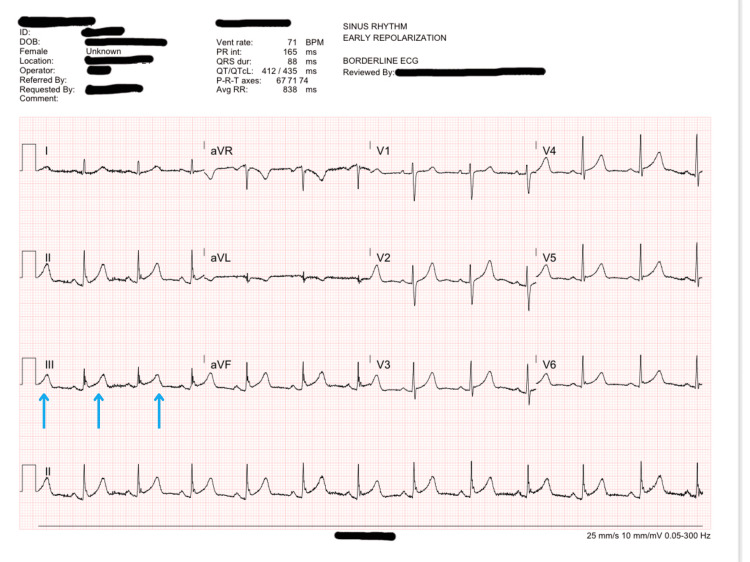
A 12-lead ECG obtained from the patient 17 minutes after the patient's arrival showed additional ST-segment elevations in lead III without clear reciprocal changes. These additional ST-segment elevations are shown in blue.

On initial evaluation, the patient’s oxygen saturation was mildly reduced at 89% on room air, and her respiratory rate was mildly increased at 22 breaths per minute. She was afebrile with a normal heart rate and blood pressure. The initial EKG obtained at the time of her arrival showed ST-segment elevations in all precordial leads and lead II and aVF (Figure 1), which raised concerns for acute coronary syndrome. The troponin level, drawn at the same time, was within the normal range, with an initial troponin I level of <0.012 ng/mL (reference range <0.034 ng/mL). Corresponding ST-segment depression was noted in aVR. A repeat EKG 17 minutes after her arrival showed additional ST-segment elevations in lead III without clear reciprocal changes (Figure 2). Apart from these findings, both EKGs were otherwise normal. 

Given the patient’s symptoms and EKG findings, ST-segment elevation myocardial infarction (STEMI) and acute pericarditis were the most likely potential diagnoses, with pericarditis remaining a consideration due to the diffuse ST-segment elevations without clear reciprocal changes and her absence of traditional cardiac risk factors. Pulmonary embolism (PE) was considered in the differential. However, bedside POCUS demonstrated no right ventricular strain, making a PE large enough to cause hypoxia less likely.

The presenting hospital did not have an interventional cardiologist, and thus, thrombolysis with tenecteplase and transfer to a tertiary facility is the standard of care for STEMI management in this setting. Due to the patient’s anticoagulation on apixaban, thrombolysis was relatively contraindicated [8]. Thus, prior to the initiation of the STEMI protocol, the cardiology service was consulted remotely. They concurred that, while transfer for PCI was necessary, fibrinolytic therapy should be considered given the transport delay. The patient received heparin, clopidogrel, atorvastatin, and aspirin, and the decision to proceed with tenecteplase was made after bedside cardiac ultrasound findings supported the diagnosis of acute OMI.

Utility of cardiac POCUS 

A bedside cardiac ultrasound was performed approximately 45 minutes after arrival by an emergency physician credentialed in cardiac POCUS. Apical four-chamber and parasternal long-axis views revealed apical wall hypokinesis with preserved basal motion, findings consistent with an anterior OMI. No pericardial effusion or right ventricular strain was observed. The cardiology consultant reviewed still images remotely and concurred with this interpretation. Tenecteplase was administered 74 minutes after arrival, approximately two hours after symptom onset. Due to adverse weather conditions, transfer to a tertiary care facility by helicopter was not possible, and the patient was transferred via fixed-wing aircraft for emergent cardiology evaluation. She left the ED approximately three hours after her arrival. 

The patient underwent PCI and was found to have an acute, severe stenosis of the proximal left anterior descending artery. Door-to-catheter-lab time was approximately 6.5 hours. A stent was placed, and a comprehensive echocardiogram (Video 1) the next day showed apical hypokinesis with preserved ejection fraction. Her initial high-sensitivity troponin at the receiving hospital was 3768 ng/L and downtrended during her stay. She was discharged three days later in stable condition, remaining functionally intact throughout her hospital course and at the 30-day follow-up. There were no bleeding complications from her tenecteplase administration.

**Video 1:** Apical four-chamber view that demonstrates a regional wall motion abnormality in the distribution of the left anterior descending artery.

## Discussion

This case exemplifies the potential of POCUS to improve care in low-resource and critical access emergency departments. In this case, cardiac POCUS identified regional wall-motion abnormalities consistent with acute OMI, which, when integrated with EKG findings, guided fibrinolytic therapy and expedited transfer for PCI. Echocardiography alone is not diagnostic for STEMI; however, it provides crucial supportive evidence that increases diagnostic confidence for an OMI.

First, POCUS reduced diagnostic uncertainty by demonstrating no pericardial effusion and by showing localized wall-motion abnormality, findings more consistent with myocardial ischemia than pericarditis. While the absence of effusion decreases the likelihood of pericarditis, POCUS alone cannot definitively rule it out. The integration of clinical context, EKG, and POCUS findings together clarified the diagnosis.

Second, POCUS served as a rapid adjunctive diagnostic tool for a time-critical condition. It identified regional wall hypokinesis while cardiac labs were still pending, thereby reinforcing the need for immediate reperfusion. Although later troponins ultimately confirmed an infarction, the early POCUS findings helped justify initiating time-sensitive treatment.

Third, POCUS is a diagnostic tool with minimal risk and exceptional accessibility. It is particularly valuable in rural and under-resourced settings where other imaging modalities or cardiology support may not be available. In this case, POCUS was the best available method to augment clinical evaluation and guide urgent decision-making.

While clinical guidelines and recommendations highlight the utility of POCUS in the diagnosis of STEMI [2, 8], there are very limited documented cases of the utilization of these recommendations [9]. Our case is, to the best of our knowledge, the first evidence of the utility of POCUS for the diagnosis of acute myocardial infarction in a rural setting. 

It is important to note that wall-motion abnormalities are not entirely specific for ischemia; myocarditis, pacing, and previous infarction can produce similar findings. Over-reliance on POCUS without integration of EKG, clinical history, and consultation may result in diagnostic error.

Recent literature and consensus guidelines continue to describe POCUS as an adjunct to, not a replacement for, standard STEMI evaluation [2, 9]. Further prospective and comparative studies on POCUS-augmented versus standard diagnostic pathways in rural STEMI care are needed to determine its true impact on time-to-reperfusion and outcomes.

Beyond this single case, broader implications include the need to expand POCUS training within emergency medicine, particularly for physicians in rural or critical-access hospitals. Developing standardized cardiac POCUS competencies could help ensure early recognition and timely coordination of care for time-sensitive cardiac emergencies.

## Conclusions

Ultimately, despite geographic barriers, point-of-care cardiac ultrasound facilitated expedited recognition and management of an acute OMI, enabling rapid access to definitive care in a setting where “time is tissue.” While causality cannot be inferred from a single case, this report illustrates how POCUS can enhance diagnostic confidence and accelerate reperfusion decision-making in rural emergency medicine. Future research should include comparative studies between POCUS-augmented and standard evaluation in STEMI care, as well as prospective evaluation of training models for rural clinicians.

This work highlights the importance of strong POCUS skills for emergency medicine physicians. Future work can be done to investigate further options for the utilization of POCUS in rural and low-resource settings and comparison trials.
